# Effectiveness of Three Agents in Pulpotomy Treatment of Permanent Molars with Incomplete Root Development: A Randomized Controlled Trial

**DOI:** 10.3390/healthcare10030431

**Published:** 2022-02-25

**Authors:** Ammar Eid, Davide Mancino, Mohammad Salem Rekab, Youssef Haikel, Naji Kharouf

**Affiliations:** 1 Department of Endodontic and Operative Dentistry, Faculty of Dentistry, Damascus University, Damascus 0100, Syria; ammarendo89@gmail.com (A.E.); msrekab@gmail.com (M.S.R.); 2 Department of Biomaterials and Bioengineering, INSERM UMR_S 1121, Strasbourg University, 67000 Strasbourg, France; mancino@unistra.fr (D.M.); youssef.haikel@unistra.fr (Y.H.); 3 Department of Endodontics and Conservative Dentistry, Faculty of Dental Medicine, University Hospital of Strasbourg, 67000 Strasbourg, France; 4 Pôle de Médecine et Chirurgie Bucco-Dentaire, Hôpital Civil, Hôpitaux Universitaire de Strasbourg, 67000 Strasbourg, France; 5 Department of Endodontic and Operative Dentistry, Faculty of Dentistry, International University for Science and Technology, Damascus 0100, Syria

**Keywords:** pulpotomy, permanent molars with incomplete root development, MM-MTA, nano-hydroxyapatite

## Abstract

The aim of this study was to investigate and compare, radiographically and clinically, the impacts of calcium-silicate based-cement (CSBC), nano-hydroxyapatite and platelet-rich fibrin (PRF) as pulpotomy agents in permanent immature molars with incomplete root development. Sixty-three participants (63 permanent immature molars) were included in this study. The patients were randomly divided into three equal groups. Fast setting MTA (MM-MTA), nano-hydroxyapatite and platelet-rich fibrin were used as pulpotomy agents. The teeth were evaluated clinically and radiographically after 6 and 12 months by two blinded examiners. Apical closure and pulp canal obliteration percentages were recorded. The in vitro reaction of the tested materials after a 7-day immersion period of the different materials in phosphate-buffered solution was analyzed using scanning electron microscopy to associate the in vitro mineralization with in vivo pulp canal obliteration percentages. Data were analyzed using Chi-square and ANOVA tests (α = 0.05). No significant difference was found between the three tested groups in terms of clinical and radiographic success (*p* > 0.05). All cases demonstrated evidence of root growth, including complete apical closure or continued apical closure. At 12 months, complete apical closure was found among the MM-MTA group (50%), nano-hydroxyapatite group (55%) and platelet-rich fibrin group (60%) (*p* > 0.05). After 12 months, pulp canal obliteration was more observed in the MM-MTA and nano-hydroxyapatite groups than in the PRF group (*p* < 0.05). MM-MTA (auto-mixed), NHA (hand-mixed) and PRF (autologous) could be used as pulpotomy agents since they exhibit comparable high clinical and radiographic success rates. However, the fact that the groups managed with MM-MTA and NHA have a higher tendency to canal obliteration might indicate that PRF should be considered the first choice material as pulpotomy agent, as it would make retreatment considerably easier.

## 1. Introduction

Although dental caries can be prevented by improving diet, limiting the ingestion of free sugars, by improving the exposure to fluoride as well as the measures of tooth brushing and facilitating access to dental care for all social classes, it is estimated that of all oral diseases, carious disease is the most common, affecting around 2.3 billion people worldwide [1,2,3,4]. Incidence of carious disease is reported to be much more frequent in pediatric patients and, as shown in different studies, to frequently affect the permanent molars, which are more susceptible to early caries soon after eruption [4,5,6]. First molars would play a key role in the healthy development of dental arches and occlusion [7].

Considering the aforementioned, the management of deep caries in permanent molars, especially those with incomplete root development, should be managed using vital pulp therapy (VPT) rather than pulpectomy or extraction. VPT allows the preservation of root dentin, apexogenesis and the preservation of mechanoreceptors, reducing the probability of tooth fracture due to overloading. Moreover, pulpectomy in many cases is not cost-effective as it is time-consuming and difficult for both patient and clinician [8,9,10,11].

In teeth with cariously or traumatized exposed pulp, of all the possible options for VPT, a pulpotomy is probably the easiest procedure to perform because microscopic visualization of the dental pulp is not mandatory for the decision-making process, and its post-operative phase is, in our experience, pain-free [12]. Pulpotomy is a reproducible procedure with a success rate of over 90% for both immature and mature teeth, provided the criteria for selecting cases are satisfied [13,14]. These criteria are based on preoperative symptoms, clinical and radiographic findings, such as the vitality of the element under consideration, the presence or absence of a radiographic lesion or even the intraoperative assessment of the color and volume of blood loss, the time required to achieve hemostasis or the biomarkers assessment in the pulpal blood and gingival crevicular fluid [15,16,17].

Different materials have been investigated, and now, there is a consensus that resin-based composites and dentine-bonding agents are contraindicated in the treatment of the exposed pulp [17,18]. The ideal pulpotomy materials should be bactericidal, biocompatible, promote root pulp cicatrization without producing excessive calcification of the remaining pulp tissue that would make re-invention very difficult and limit tooth discoloration. Histological and clinical outcomes have demonstrated that calcium silicate-based cements should be preferred to calcium hydroxide in the treatment of exposed pulp, including full pulpotomy [19,20,21]. However, not all calcium silicate-based cements are the same, and today, there is a wide range of these available with similar biological properties but different chemical compositions that influence their radiopacity, setting time, pigmentation potential on the tooth and their immunomodulatory properties that affect their biocompatibility, osteogenicity and bioactivity [22,23]. Moreover, to manage pulpotomy, except calcium hydroxide and calcium silicate cements, other biomaterials, including platelet-rich fibrin (PRF) and calcium-enriched mixture [24], could be used.

Platelet-rich fibrin (PRF) [11] is an autologous source of platelets that is enriched with several growth factors. PRF, firstly developed by Choukroun et al. [25], is an autologous source of platelets that is enriched with several growth factors. Because of its autologous nature, it provokes little or no inflammatory response when in contact with the pulp [8,10,11,14,26,27]. However, it may not be a procedure accessible to all general practitioners (GPs) because of the need of bloodletting a patient and having a centrifuge.

Nano-hydroxyapatite (NHA), another biocompatible and bioactive biomaterial, is an attractive inorganic material for endodontic application thanks to its chemical composition, biomimetic and nanoscale topography and its positive proliferative action on several mesenchymal cell types [28,29,30]. This biomaterial was used in humans as a pulp capping agent [31,32] and in pigs as pulp capping and pulpotomy material [33]. These studies [32,33] reported that nano-hydroxyapatite is a promising material as a direct pulp capping agent due to its ability to produce favorable cellular, vascular response and complete dentinal bridge formation. However, the dentinal bridges formed in these cases are tubular [33], which could indicate a repair process that occurred from pulpal fibroblasts producing calcified scar tissue without odontoblast differentiation [34].

Calcium silicate-based cements (CSBCs), as stated above, are widely used in the pulpotomy technique [35,36]. They have several biological and physicochemical properties, such as antibacterial effects, calcium ion releasing, low solubility and good sealing ability [37,38]. Although they have been extensively studied and have excellent potential as a pulpotomy material, several cases of discoloration have been reported [39,40]. These materials are available in manual mixing or auto-mixing [41]. However, manual mixing could impact the physicochemical properties of these calcium silicate-based cements [42,43]. Kharouf et al. [43] reported that auto-mixing CSBCs demonstrated a homogeneous mixture with lesser voids in the material structure and no alteration of the powder/liquid ratio. MM-MTA (MicroMega, Besançon, France) is a pre-dosed capsule that allows a stable powder/liquid ratio and can be directly delivered in the treated location through the nozzle of the capsule [44]. The addition of CaCO_3_ in its formulations makes this cement a fast-setting CSBC.

The choice of pulpotomy agents could play an important role in the clinical outcome and therefore should be made on the basis of clinical and histological evidence with considerations based primarily on the preservation of pulpal viability over time [24].

To the best of our knowledge, to date, no study has assessed the use of NHA as a pulpotomy agent in the treatment of permanent molars with incomplete root canal development in humans.

Therefore, the aim of this study was to evaluate both the radiographic and clinical effectiveness of MM-MTA, NHA and platelet-rich fibrin used as pulpotomy agents in permanent molars with incomplete root development. The null hypothesis tested was that the use of different pulpotomy agents would influence the clinical and radiographic outcomes of immature permanent molars with incomplete root development.

## 2. Materials and Methods

### 2.1. Study Design

**Figure 1:**
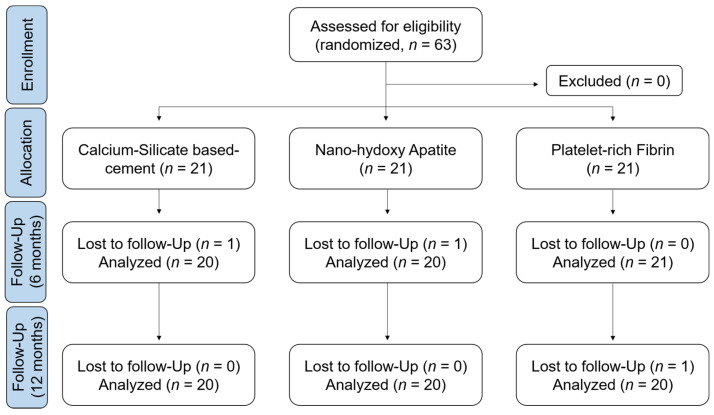
A CONSORT diagram showing the flow of participants through each stage of the study.

This randomized clinical trial study was conducted at the faculty of dental medicine in Damascus University-Syria, between February 2020 and November 2021. The protocol was approved by the Ethics Committee at the Ministry of Higher Education in Syria (1898/SM-3962), and the study adhered to the ethical values of the Declaration of Helsinki. A randomized parallel-group controlled clinical trial study was designed in accordance with the CONSORT guidelines (Figure 1) to ensure the quality and transparency of this study.

### 2.2. Study Participants

Study children were recruited from the cohort of patients referred to the Department of Endodontic Dentistry. Children agreed to participate in the research study and written informed consent from their parents was provided. A total of sixty-three permanent molars were included in the study.

Inclusion criteria were as follows:

- Children aged between 6 and 12 years;
- Symptomatic/Asymptomatic vital permanent molars with deep caries lesion close to the pulp chamber roof or with clinical carious exposure of the pulp after spoon excavation or with pulp exposure after trauma (within 48 h) and the existence of bleeding upon exposure;
- Incomplete root development visible in the radiograph;
- Intraoperative bleeding time of radicular pulp not exceeding 5 min;
- Physiological mobility;
- Restorable crown.

Non-inclusion criteria were as follows:

- History of systemic disease or allergic reaction;
- History of spontaneous pain;
- Presence of external or internal resorption;
- Apical or inter-radicular lesion.

### 2.3. Study Intervention

The patients were randomly divided into three treatment groups using an online software at www.randomizer.org (accessed on 1 May 2020). The block randomization method was performed to obtain balanced and equal groups. A fully trained operator performed all clinical steps to avoid interoperator variables and did not decide which teeth went to which tested group. Cases fulfilling the inclusion criteria to perform pulpotomy treatment were managed in strict accordance with the American Academy of Pediatric Dentistry guidelines [45]. The nature and risks of each treatment option were explained to the parents of children, and they were also blinded to the used material. MM-MTA (MicroMega, Besançon, France) was used in group 1 (G1), nano-hydroxy apatite (Ghimas, Casalecchio di Reno, Italy) was performed in group 2 and platelet-rich fibrin was performed in group 3 (G3).

**Figure 2:**
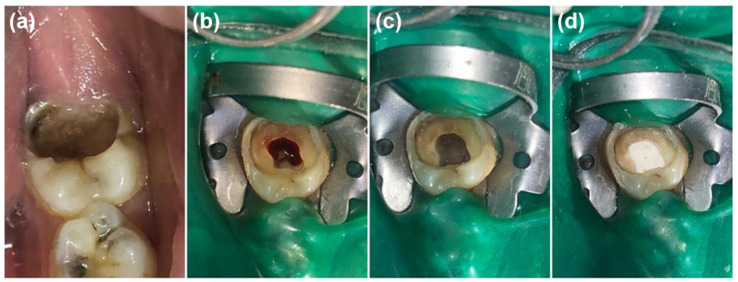
(**a**) Tooth isolated with rubber dam; (**b**) caries and coronal pulp removal; (**c**) pulp stumps after achieving hemostasis; (**d**) the placement of the pulpotomy agent.

Clinical and radiographic examinations were carried out for all participants. A preoperative radiographic assessment with a standardized bisecting angle method was performed using periapical films (Vatech ez sensor, Gyeonggi-do, Korea). An inferior alveolar nerve block with supplemental buccal infiltration using 2% lidocaine (for a total of 3.6 mL) with 1:80,000 epinerphrine (New Stetic S.A, Guarne, Colombia) was administrated for each tooth. After isolation using a rubber dam (Figure 2a), all cavities and coronal caries were prepared and removed using a round red bur (Dentsply Maillefer, Tulsa, OK, USA), and the last carious dentin overlying the pulp was left, and the resultant cavity was investigated for pulpal exposure. The coronal access to the exposed pulp chamber was performed by a high-speed bur (Dentsply Maillefer, Tulsa, OK, USA) and copious sterilized normal saline. The coronal pulp amputation was performed using a sharp spoon excavator (Figure 2b). In order to achieve hemostasis (Figure 2c), sterilized cotton pellets moistened with sterile saline was placed with slight pressure over the pulp’s stumps for 2–3 min. If the bleeding could not be controlled after 5 min of cottons pellets removal, the tooth was excluded from this study [46].

**Figure 3:**
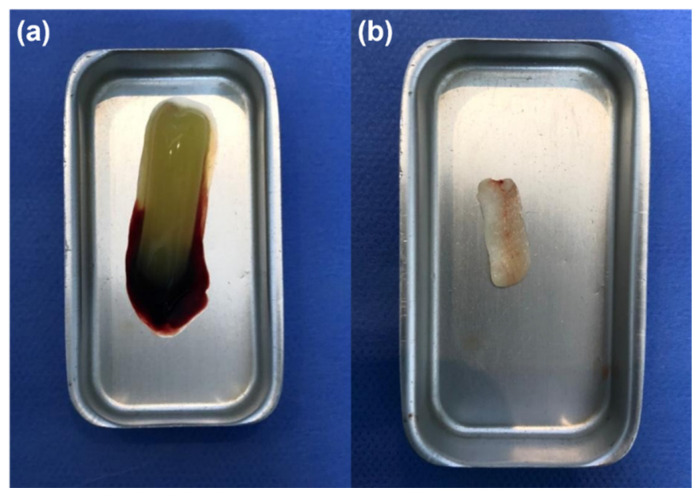
(**a**) The PRF clot; (**b**) The PRF membrane.

MM-MTA auto-mixed CSBC “Bioceramics” (G1) was applied directly, after achieving hemostasis, on the pulp stumps and condensed lightly with an amalgam plugger, using the coronal part of sterile paper points to achieve 2–3 mm thickness. NHA (G2) was mixed with a sterilized physiological solution until it reached the ideal consistency (a powder/liquid ratio of 3:2) for 30 s. A putty consistency was obtained, then placed on the pulp stumps and condensed lightly using the previous procedure to achieve 2–3 mm thickness. After 10 min of placing the materials, thick intermediate restorative material cement (powder/liquid ratio of 1:2) (IRM; 2–3 mm) (Dentsply Sirona, PA, USA) was used to fill the pulp chamber. For the third group, a freshly prepared PRF membrane was placed on the pulp stumps, and the coronal pulp chamber was filled directly with a thick mix of zinc oxide-eugenol cement. Concerning the preparation steps of PRF, 5 mL of blood were withdrawn from the patient and put without an anticoagulant in a 10 mL test tube. The samples were immediately centrifuged using a tabletop centrifuge (Hettich, Hohberg, Germany) at 3000 revolutions per minute for 10 min. After centrifuging, the PRF clot was collected and squeezed between the sterile dry gauges to drive out the fluids trapped in the fibrin matrix and to obtain a resistant autologous membrane (Figure 3). Directly, thick intermediate restorative material cement (powder/liquid ratio of 1:2) (IRM; 2–3 mm) (Dentsply Sirona) was used to fill the pulp chamber. After the application of different materials, all the teeth were restored by direct composite restoration; otherwise, amalgam (DPI Alloy, Dental Products of India, Mumbai, India) restoration followed with a stainless steel crown (UnitekTM, 3MTM ESPETM, Minneapolis, MN, USA). After treatment, a periapical radiograph was immediately taken. The dentist recommended the patients and their parents to signal any pain or reactions related to the treatment within next 7 days, and paracetamol was prescribed if the participants was in pain.

### 2.4. Study Follow-Up and Evaluation

The patients were recalled for clinical and radiographic evaluations after 6 and 12 months of treatment. Two examiners (experienced endodontists) blinded to all the experimental groups assessed the teeth clinically and radiographically by evaluating the root development, interdental and inter-radicular areas and root canal space. When different evaluations were attributed by the two examiners, they reevaluated the case with a third examiner to reach a consensus. Clinical success has been considered when the patients have no pain, either spontaneous or induced by thermal or percussive stimuli; no abscess or fistulation; no sinus tract; no swelling; no pathological tooth mobility; and no tenderness associated with the tooth [10,11,47]. Moreover, radiographic success has been considered when there are no internal/external resorption, no bone destruction, no periapical lesions and no periodontal ligament widening. The radiographic evidence of root growth and canal obliteration was also considered.

### 2.5. Statistical Analysis

Before recruitment, the sample size was calculated using Minitab (Minitab^®^ 20 software L.L.C., PA, USA). Three groups of 21 teeth each were finally formed in order to have 80% power and an alpha error probability of 0.05. Statistical analysis was performed using the SPSS program version (SPSS Inc., Chicago, IL, USA). A chi-square test was used to determine whether significant differences existed in the complete apical closure and canal obliteration at 6 and 12 months. One-way analysis of variance test was used to determine whether significant differences existed in the age of participants between different groups. In all tests, a statistical significance level of α = 0.05 was adopted. Cohen’s Kappa test was applied to verify the agreement between the two observers using Minitab software (Minitab^®^ 18.1, Minitab, Inc., Pennsylvania State University, State College, PA, USA).

### 2.6. Scanning Electron Microscopy Analysis (SEM)

After the end of trial (12 months), PRF, NHA and MM-MTA samples were observed using SEM (Quanta 250 FEG scanning electron microscope “FEI Company, Eindhoven, The Netherlands”; 10 kV acceleration voltage of the electrons). In addition, six samples of each material were used. MM-MTA and NHA were placed into Teflon molds (internal diameter: 3 mm and height: 3.8 mm) and stored in the dark in a container at 37 °C for 48 h to achieve a proper setting time. The PRF membrane was cut to achieve a diameter of 3–5 mm and a thickness of 1–3 mm. Three samples of each material were immersed in phosphate-buffered saline (PBS10x, Dominique Dutscher, Bernolsheim, France) at 37 °C for 7 days. PRF samples were fixed by using a solution of 0.05 M glutaraldehyde in 4% cacodylate buffer for 8 h. After that, the samples were rinsed using a 4% cacodylate buffer three times, 5 min each. The samples were dehydrated in graded series of ethanol (35%, 50%, 70%, 95% and 100%) for 3 min each. After the graded series of ethanol solution, the samples were dried using a chemical drying agent, Hexamethyldisilazane (HMDS). The samples were transferred from 100% ethanol into 1:1 solution of HMDS for 10 min, then transferred into 100% HMDS two times, 10 min each [48]. All specimens were sputter-coated with gold–palladium (20/80) using a Hummer JR sputtering device (Technics, CA, USA) and analyzed at a magnification of 3000× and 12,000× for morphological changes and mineralization processes through SEM.

## 3. Results

Sixty-three patients were included in the present study and received pulpotomy treatment with different materials. During the trial, three patients were lost in the follow-up visits (one patient in each group). Finally, twenty patients from each group were clinically and radiographically evaluated in a 12-month follow-up. No significant difference was found between the mean ages of patients (Table 1) or between the sex of the patients in each group (*p* > 0.05). As age and sex could influence treatment prognosis, we recorded these preoperative data following a binary classification, considering for the variable age, patients up to 8.5 years old and patients older than 8.5 years old, and for the variable sex, considering female or male patients. In our study, age and sex do not appear to influence treatment outcome.

**Figure 4:**
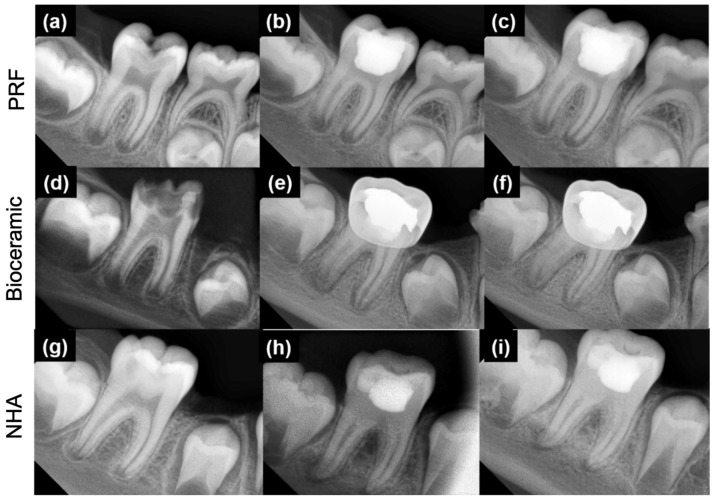
(**a**) Preoperative radiograph of an immature mandibular molar; (**b**) no complete apical closure at 6 months; (**c**) no complete apical closure at 12 months “continued apical closure”; (**d**) preoperative radiograph of an immature mandibular molar; (**e**) no complete apical closure at 6 months “continued apical closure”; (**f**) complete apical closure at 12 months; (**g**) preoperative radiograph of an immature mandibular molar; (**h**) complete apical closure at 6 months; (**i**) complete apical closure at 12 months.

During the follow-up appointments, none of the patients in the three tested groups had swelling, pathological tooth mobility, abscess, fistulation and pain. Moreover, no radiographic pathological signs such as root resorption, inter- and peri-radicular bone destruction and periodontal ligament widening were reported. For all clinical observations, a perfect agreement was reported between the two examiners. For the radiographic assessments, the Cohen’s kappa value for interobserver agreement of all groups was 0.91. No statistical difference was found among the three groups regarding apical closure at 6 and 12 months (Table 1). In all groups, three radiographical types were observed during the follow-up (Figure 4): (1) No complete apical closure at 6 and 12 months; (2) no complete apical closure at 6 months “continued apical closure” and a complete apical closure at 12 months; and (3) a complete apical closure at 6 and 12 months.

Pulp canal obliteration was more prominent in groups 1 and 2 (MM-MTA and nano-hydroxyapatite) at 6 months without statistical difference (*p* = 0.111) and 12 months with statistical difference (*p* = 0.014) when comparing their radiographic results with group 3 (PRF) (Table 1).

**Figure 5:**
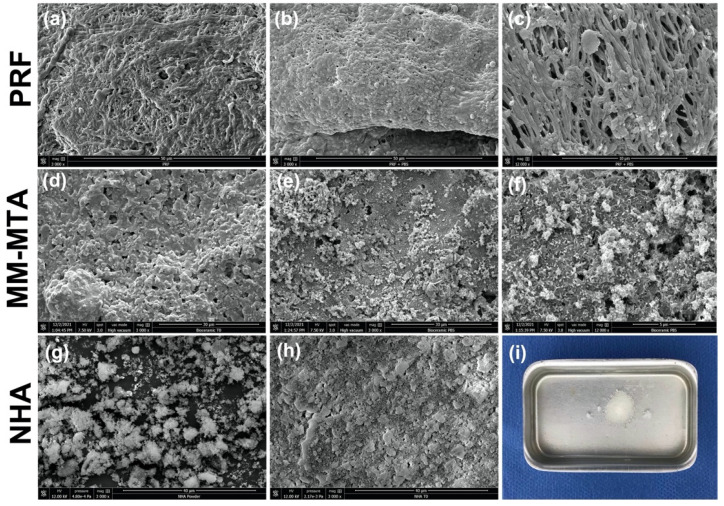
Representative scanning electron microscope images at 3000× magnification (**a**,**b**,**d**,**e**,**g**,**h**) and 12,000× magnification (**c**,**f**). The morphology observed for (**a**) PRF and (**d**) for MM-MTA and (**h**) NHA before immersion in PBS; (**g**) NHA powder; (**b**,**c**) the morphology observed for PRF, (**e**,**f**) for MM-MTA and (**i**) for NHA after immersion in PBS for 7 days at 37 °C.

After the pulp canal obliteration findings, SEM analyses were performed to observe the morphological and mineral deposition changes on each material’s surface in contact with PBS in order to evaluate the mineral deposition rate, which could be related with canal obliteration process. The results of the tested materials’ surfaces before and after immersion in PBS are described in Figure 5. Crystallites were observed on the MM-MTA surfaces after 7 d of immersion. The most observed crystallites were urchin and globular crystalline microstructures (Figure 5e,f). In contrast, the PRF membrane demonstrated no mineralization process, and no crystallites were observed on its surface after the immersion period (7 days) (Figure 5b,c). Concerning NHA samples, after the immersion period, NHA was soluble in PBS (Figure 5i).

## 4. Discussion

Immature permanent teeth present a fragile root with open apices. The primary aim of vital pulp treatment of these teeth is to ensure the complete root apex formation and reinforcing the root walls [45,49]. Pulpotomy, a type of VPT, is considered the suitable procedure for an immature permanent tooth with exposed pulp due to a deep caries or trauma [8,9,10,11,36]. Different pulpotomy dressing agents are being used in the pulpotomy procedure to manage incomplete root development teeth [13].

The present study aimed at assessing the outcome of three different pulpotomy dressing agents in immature permanent molars. In the present study, teeth crowns were restored using only resin-composite or amalgam followed by a stainless steel crown. These materials were used according to the damage degree of the crown. No statistical difference was found between the tested pulpotomy agents (*p* > 0.05) based on clinical analysis, whereas, radiographically, the group managed with PRF showed statistically lower tendency to pulp canal obliteration when compared to the remaining two groups (*p* < 0.05). No statistical difference in terms of canal obliteration was noted between the groups using NHA and MM-MTA (*p* > 0.05). Therefore, the null hypothesis could be partially rejected. Our finding showed, at 6 and 12 months, that the three biomaterials could be used as pulpotomy agents to ensure the continuity of the root canal. Although there was no difference in terms of results among the three materials tested in our study, their mechanism of action appears to be quite different.

PRF, similar to other platelet concentrates, is highly biocompatible due to its autologous nature, modulating the inflammatory response when brought into contact with pulp tissue through the release of healing cytokines and different growth factors [11,50,51,52,53,54]. In addition, Choukroun’s PRF, unlike other platelet concentrates, dissolves much more slowly and its fibrin matrix is remodeled very slowly, increasing its healing capacity [26].

**Figure 6:**
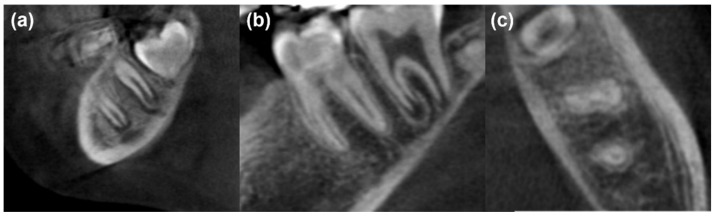
Cone-beam computed tomography (CBCT) analysis for some teeth after 12 months. (**a**) Sagittal view of incomplete apical closure in pulpotomy treatment using PRF; (**b**) sagittal view of complete apical closure in pulpotomy treatment using PRF; (**c**) axial view of complete apical closure in pulpotomy treatment using PRF.

The present study showed that the use of PRF membrane could result in incomplete apical closure (19.04%, Figure 6a) at 6 months and complete apical closure (60% of cases) after 12 months (Figure 6b,c). In accordance, Keswani et al. [11] reported a complete root closure of 65.5% and 88.8% at 12 and 24 months, respectively, in permanent molars with incomplete root development. The same authors reported, in accordance with our results for MM-MTA, a complete root development using mineral trioxide aggregate (MTA) in 51.8% of cases after 12 months [11]. These results are due to the properties of CBSCs and, in particular, to their biocompatibility, antimicrobial activity, bioactivity, angiogenetic and osteogenetic activity [55]. In addition, using an auto-mixed CBSC would be advantageous because it avoids errors during mixing that could affect their physical and chemical characteristics [43].

Nanomaterials have become a very attractive material for biomedical application [30]. The biocompatibility and structural similarity of nano-hydroxyapatite to bone and tooth increase the interest of using this material in orthopedics and dentistry. Although NHA is already used in different branches of dentistry, our study is the first to assess the use of NHA as a pulpotomy agent in the treatment of permanent molars with incomplete root canal development in humans. The present study demonstrated equal clinical and apical closure results of nano-hydroxyapatite comparing to PRF and MM-MTA (*p* > 0.05). These findings should be linked to NHA’s ability to enhance hard tissue regeneration, odontoblast-lime and osteoblast cells migration [32,33], despite an increased inflammatory cell response and cell necrosis in the pulp tissue adjacent to the capping material compared to CSBCs [32]. The advantage of using NHA as a pulpotomy agent is its good handling compared to PRF handling and preparation. Moreover, the chemical composition of hydroxyapatite is similar to the mineral phase of the bone and dentin [56], whilst MM-MTA contains aluminum and bismuth and other products that could alter the cell viability. The NHA product is less expensive than MM-MTA comparing the quantity of each material; however, PRF, an autologous material, stays less expensive, but it requires lot of steps.

Pulp canal obliteration was more announced in the MM-MTA (15%, 35%) and nano-hydroxyapatite (20%, 45%) groups than the PRF (0%, 5%) group at 6 and 12 months, respectively. In accordance, Nasrallah et al. [57] reported 54% of pulp canal obliteration after using Biodentine (calcium silicate-based cement) in pulpotomy treatment at 12 months. Moreover, Rajasekharan et al. [58] reported high pulp canal obliteration percentages for CSBC. In accordance with our results, several studies [10,59] showed no canal obliteration among PRF pulpotomy treatment, whilst Hiremath et al. [54], in a clinical case of permanent molar with pulpitis, noted an obliteration after 22 months of pulpotomy treatment with PRF. However, in the present study, pulp canal obliteration was not considered a failure. However, if we speculate, since one of the main characteristics that an endodontic filling material should have is to easily guarantee a possible re-intervention in the case of failure [60], a biomaterial that limits root canal obliteration should be preferred. The high releasing of Ca^2+^ from the CSBCs could be related to its ability to induce tissue repair and to stimulate mineralization on the CSBCs’ surface, resulting in an inevitable canal obliteration [61]. For the nano-hydroxyapatite material, several studies showed that this material encourages hard tissue and osteodentin formations [32,33]. In order to associate the findings of pulp canal obliteration rates with the pulpotomy agents used, SEM analyses were performed on each material surface after a period of immersion in PBS at 37 °C. PBS solution was used to mimic the in vivo dental tissue fluids [62] in order to investigate, in a simplified approach, the mineral changes that could occur on the material surfaces. SEM observations demonstrated crystallite formation onto MM-MTA surfaces, whilst no mineralization procedure and no changes were observed for PRF surfaces after immersion for 7 days in PBS. Furthermore, CSBCs are able to form calcium silicate hydrate and calcium hydroxide once it is in contact with a humid environment [41,43,63]. These crystallite formations could be related with the higher pulp canal obliteration percentages that were observed among MM-MTA and NHA groups. In contrast, SEM analyses, after 7 days in PBS, were not performed for the NHA group due its high solubility, which prevents SEM preparation and observations. PRF does not demonstrate any mineral deposition on its surface, which could be related with none or low (5% after 12 months) pulp canal obliteration percentage within this group.

One of the limitations of PRF was the potential drawback of collecting the required amount of blood from the participants along with the need for special materials to perform a good fibrin membrane. Another limitation of the PRF material was the difficult application compared to the application of nano-hydroxyapatite and auto-mixed bioceramic materials. The mixing of nano-hydroxyapatite and the potential of making an error concerning the powder/liquid ratio was a limitation of this material. The high solubility rate of NHA in PBS was a limitation for this study. In contrast, the good handling and facility of application of the auto-mixed bioceramic material give this material an advantage in using it in pulpotomy treatment.

Furthermore, the chemical interaction between the CSBCs and pulp cells deserves further investigation. Therefore, future studies should be performed to evaluate the solubility, mechanical properties and filling ability of the nano-hydroxyapatite material. Moreover, additional studies on cytotoxicity are recommended in order to analyze the biocompatibility of the nano-hydroxyapatite and bioceramic materials.

## 5. Conclusions

The three biomaterials used in this study, MM-MTA, NHA and PRF, as pulpotomy agents have shown a high rate of clinical and radiographic success since they allowed the root development, including complete apical closure or continued apical closure, in immature permanent mandibular molars. However, the group managed with PRF (autologous) showed a statistically lower tendency to pulp canal obliteration when compared to the MM-MTA (auto-mixed) and NHA group (manually mixed) (*p* < 0.05). This data could be decisive in the choice of pulpotomy agent and might indicate that PRF should be considered the first choice material as a pulpotomy agent, as future eventual retreatment, in the case of failure, could be considerably easier.

## Figures and Tables

**Table 1:** Age, sex and radiographic evidence of root canal growth in the three tested group at 6- and 12-month follow-ups. * *p* < 0.05.

Criteria/Group	MM-MTA (G1)	Nano-Hydroxyapatite (G2)	PRF (G3)	Statistical Analysis
Age (years)	8.6 ± 2.0	8.8 ± 1.8	8.8 ± 2.1	*p* = 0.911
Sex females (%)	11 (55)	10 (50)	9 (45)	*p* = 0.819
Apical closure complete (6 months) (%)	5 (25)	3 (15)	4 (19.04)	*p* = 0.726
Apical closure complete (12 months) (%)	10 (50)	11 (55)	12 (60)	*p* = 0.817
Canal obliteration (6 months) (%)	3 (15)	4 (20)	0 (0)	*p* = 0.111
Canal obliteration (12 months) (%)	7 (35)	9 (45)	1 (5)	*p* = 0.014 *

## Data Availability

Not applicable.
